# A Multiple Stimuli–Responsive Ag/P/S Complex Showing Solvochromic and Mechanochromic Photoluminescence

**DOI:** 10.3390/molecules28145513

**Published:** 2023-07-19

**Authors:** Jia-Jun Yan, Yu Wu, Weijia Zhai, Ningwen Yang, Hong-Xi Li, Wei Yang, Chengrong Lu, David James Young, Zhi-Gang Ren

**Affiliations:** 1 Suzhou Key Laboratory of Novel Semiconductor-Optoelectronics Materials and Devices, College of Chemistry, Chemical Engineering and Materials Science, Soochow University, Suzhou 215123, China; 2 Faculty of Food Science and Technology, Suzhou Polytechnic Institute of Agriculture, Suzhou 215008, China; 3 Glasgow College UESTC, University of Electronic Science and Technology of China, Chengdu 611731, China

**Keywords:** Ag/P/S complex, stimuli-responsive material, solvochromic luminescence, mechanochromic luminescence

## Abstract

The reaction of CF_3_COOAg, 3-bdppmapy (*N*,*N*-bis(diphenylphosphanylmethyl)-3-aminopyridine) and HTZ (1,2,4-triazole-3-thiol) in CH_2_Cl_2_/MeOH resulted in a dinuclear Ag/P/S complex [Ag_2_(TZ)_2_(3-bdppmapy)_2_]·*x*Sol (**1**·*x*Sol). Crystals of **1**·*x*Sol converted to **1**·2MeOH in air at room temperature and further to **1** under vacuum upon heating. The solid-state, room-temperature photoluminescent emission of **1**·*x*Sol (510 nm) shifted to 494 nm (**1**·2MeOH) and 486 nm (**1**). Grinding solids of **1**·2MeOH in air resulted in amorphous **1G** characterized by solid-state emission at 468 nm, which converted to **1GR** with 513 nm emission upon MeOH treatment. Grinding **1GR** in air returned **1G,** and this interconversion was reproducible over five cycles. The solid-state photoluminescence of **1G** changed in response to vapors containing low–molecular weight alcohols but remained unchanged after exposure to other volatile organic compounds (VOCs) or to water vapor. Test papers impregnated with **1G** could detect methanol in vapors from aqueous solutions at concentrations above 50%. Complex **1G** is, therefore, an example of a stimuli-responsive molecular sensor for the detection of alcohols.

## 1. Introduction

Alcohols are an important class of organic reagents with plentiful applications in food, pharmaceutical, and chemical industries. Low–boiling point alcohols are prone to form vapors that usually exhibit significant impacts on human health. In particular, high-concentration methanol (MeOH) can cause damage to the human nervous system and even lead to death [1,2]. Therefore, the rapid detection of MeOH is of great importance to environmental science. Compared to other common detection methods, photoluminescent sensors have gained interest from researchers as they show various advantages, including cost-effectiveness, high sensitivity, and simple operation. The detection mechanism has been reported mainly within the intermolecular interactions between the MeOH molecule and the major structures of the sensor materials [3,4]. Stimuli-responsive photoluminescent materials have been intensively investigated as smart materials with potential applications, such as sensors and information materials [5,6,7,8,9,10]. Many stimuli-responsive materials allow the tuning of performance and function toward different external stimuli [11,12,13,14,15,16]. Silver(I) complexes have been developed as photoluminescent stimuli-responsive materials with diverse structures and strong emissions [17,18,19]. External stimuli include chemical vapor [20,21,22,23], heat treatment [24,25,26], and mechanical force [27]. Inter- and intra-molecular interactions are likely affected by these stimuli, resulting in changes to emission color or intensity. Consistent with the hard and soft acid–base (HSAB) concept, P and S atoms can form stable coordination bonds with Ag(I) ions. This coordination affects the charge transfer properties of the complex, potentially increasing its luminescence. Other functional groups, such as phenyl, alkyl, and amino groups, may form non-covalent interactions, including π···π, C−H···π, and hydrogen bonds [28,29,30], which influence the rigidity of the structures, suppress non-radiative transitions, and improve luminescence lifetimes [31,32,33]. Koshevoy et al. reported that a series of Cu(I) and Ag(I) thiocyanate complexes containing phosphine ligands showed efficient thermally activated delayed fluorescence (TADF) [34]. Phosphine and SCN ligand affect not only the nuclearity of products but also the characteristics of excited states.

We focused on the design and photoluminescent performance of Ag/P complexes [35] and synthesized a family of such compounds with stimuli-responsive luminescence bearing P/N [36], P/C≡C [37], and P/S [38] hybrid ligands. For instance, a coordination polymer [Au_4_(dppmt)_4_(AgCl)_2_]*_n_* with the P/S hybrid ligand dppmtH ((diphenylphosphino)methanethiol) showed selective and reversible vapor-chromic response toward MeOH [39]. Herein, we report the preparation of a dinuclear Ag(I) complex [Ag_2_(TZ)_2_(3-bdppmapy)_2_]·*x*Sol (**1**·*x*Sol) by the reaction of CF_3_COOAg with a diphosphine ligand 3-bdppmapy (*N*,*N*-bis(diphenylphosphanylmethyl)-3-aminopyridine) and a S/N hybrid ligand HTZ (1,2,4-triazole-3-thiol). The gradual removal of the MeOH molecules from **1**·*x*Sol resulted in **1**·2MeOH, which exhibited solvochromic and mechanochromic photoluminescence.

## 2. Results and Discussion

### 2.1. Synthesis and Characterization

**Figure 1:**
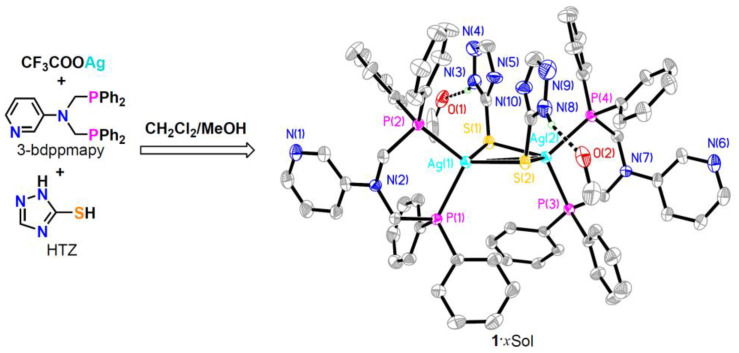
Synthesis and crystal structure of **1**·*x*Sol with 50% thermal ellipsoids. All hydrogen atoms except for those of the N−H···O bonds are omitted for clarity.

Compound **1**·*x*Sol was isolated as single crystals by the reaction of CF_3_COOAg, 3-bdppmapy, and HTZ (molar ratio of 1:1:1) in CH_2_Cl_2_/MeOH, followed by slow diffusion with hexane (Figure 1).

Single-crystal X-ray diffraction (SCXRD) analysis of **1**·*x*Sol at 120 K revealed that it crystallized in the triclinic *P*ī space group. Each asymmetric unit contained one [Ag_2_(TZ)_2_(3-bdppmapy)_2_] molecule and two MeOH solvent molecules. As shown in Figure 1, each Ag(I) atom was tetrahedrally coordinated with two S atoms from two TZ^−^ anions and two P atoms from one cleating 3-bdppmapy ligand. The Ag1−Ag2 distance of 3.3076(5) Å was shorter than the sum of van der Waals radii of two Ag atoms (3.44 Å), indicating the existence of a weak argentophilic interaction. Each TZ^−^ anion bridged two Ag(I) atoms, thus forming a quadrilateral Ag_2_S_2_ plane. The bond lengths of Ag1−S1, Ag1−S2, Ag2−S1, and Ag2−S2 were 2.6388(10), 2.5856(10), 2.6173(10), and 2.5932(10) Å, respectively. The triazole groups of the two TZ^−^ anions extended to the same side of the Ag_2_S_2_ plane.

**Figure 2:**
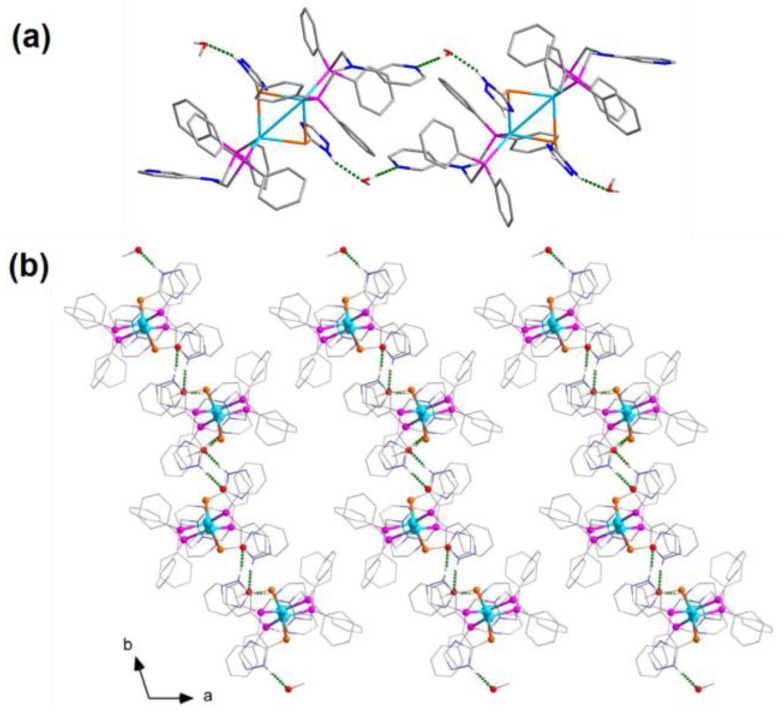
(**a**) The hydrogen bonds (dashed lines) in **1**·*x*Sol. (**b**) Packing of molecular arrays in **1**·*x*Sol viewing along the *c* axis. All hydrogen atoms except for those of the N−H···O and O−H···N bonds are omitted.

Only two MeOH molecules were successfully located from the Fourier map and connected to the major structure via hydrogen bonds (Table 1 and Figure 2a). There was no coordination interaction between Ag(I) atoms and these MeOH molecules. Other solvent molecules were not located at appropriate positions. Upon treating the data with the SQUEEZE (in PLATON v1.18) program, a large void with a volume of 1237 Å^3^ was found in each cell (Figure 2b), which was about 30% of the total cell volume.

**Figure 3:**
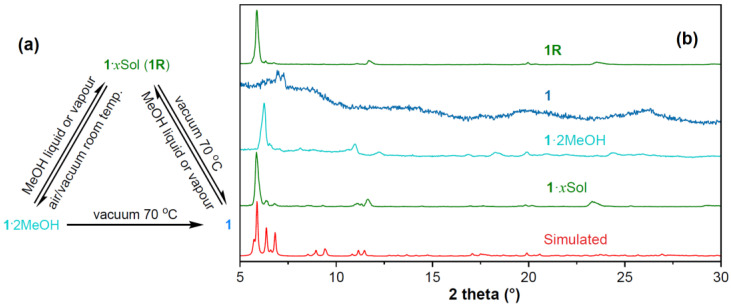
(**a**) Interconversions and (**b**) PXRD patterns of **1**·*x*Sol, **1**·2MeOH, **1**, and **1R**.

The PXRD pattern of as-synthesized **1**·*x*Sol fit well with that simulated from the SCXRD data (Figure 3). The TGA curve of **1**·*x*Sol in a stream of N_2_ (Figure S1, Supplementary Materials) indicated that all lattice solvent molecules escaped over the range of ambient temperature to 100 °C, while the major structure remained stable until 150 °C. The theoretical electron number (406 electrons in the lattice voids of each cell) reported by the SQUEEZE program matched 11.3 MeOH molecules relative to each major structure [40,41]. Because the desolvation of the solvent molecules at room temperature was quite fast, the TGA measurement of **1**·*x*Sol (Figure S1) could not give an accurate *x* value. We, therefore, measured the weights of a portion of as-synthesized **1**·*x*Sol before and after vacuum treatment at room temperature. With the rapid desolvation, the weight losses in several repeated experiments fluctuated between 21% and 17%, which correlated to 12 and 9.4 MeOH molecules, respectively. Thus, the solvent was speculated to be 11.4–14 MeOH molecules upon considering the 2 hydrogen-bonded MeOH molecules. When **1**·*x*Sol was left in air for more than one day or under vacuum for 0.5–12 h at ambient temperature, the TGA curve of the resulting solid showed a weight loss of 3.9% below 100 °C, which matched with the removal of two MeOH molecules (Calcd 4.4%), suggesting a molecular formula of **1**·2MeOH. Based on the SCXRD results, these two residual MeOH molecules are most likely the two hydrogen-bonded solvates. The completely desolvated compound **1** was prepared by heating **1**·*x*Sol or **1**·2MeOH at 70 °C under vacuum for more than 2 h.

The rapid desolvation of **1**·*x*Sol was accompanied by obvious crystal cracking. N_2_ sorption measurements for crystals of **1**·2MeOH and **1** revealed that their BET surface areas were nearly zero, which indicated the original void structures of **1**·*x*Sol had totally collapsed when the solvent molecules escaped. We supposed that because there was no other obvious intermolecular interaction between the major structures except for the aforementioned hydrogen bonds, the large voids in **1**·*x*Sol were only propped open by the solvent molecules and thus were quite unstable. In addition, the PXRD pattern of **1**·2MeOH showed it was crystalline (Figure 3), whereas that of **1** indicated it was nearly amorphous. These significantly changed patterns indicated that stepwise phase transitions occurred when the solvent molecules were gradually eliminated from the lattice voids. Sample **1R** was generated by immersing crystals of **1** in a drop of liquid MeOH or exposure for several minutes to a stream of air saturated with MeOH and showed a similar TGA weight loss (Figure S1) and PXRD pattern to those of **1**·*x*Sol, indicating that the phase transitions were reversible.

Solids of **1**·2MeOH were stable in air and moisture; soluble in DMF and DMSO; slightly soluble in CH_2_Cl_2_ and acetone; and insoluble in Et_2_O, hexane, and water. The IR spectrum of **1**·2MeOH (Figure S2) revealed that vibration related to the S−H group at 2613 cm^−1^ in HTZ disappeared, while the stretching vibration of the triazole group at 1557 cm^−1^ shifted to 1670 cm^−1^, indicating the thiol group was deprotonated during the synthesis. The ^1^H NMR (in DMSO-*d_6_*, Figure S3) spectrum contained signals for the −CH_3_ (3.50 ppm, 6H), −CH_2_− (4.85 ppm, 8H), −Ph, −Py, and TZ^−^ (6.54–7.92 ppm, 52H). The ^31^P NMR (in DMSO-*d_6_*, Figure S5) spectrum consisted of a signal centered at −9.19 ppm attributed to −PPh_2_. **1**·2MeOH was stable toward natural light, while the photosensitivity experiment by placing the solids under a 365 nm LED (0.5W, light area 0.8 × 0.8 cm^2^) indicated it remained unchanged in the first 15 min, whereas it turned yellow and gray after longer irradiation (0.5 to 15 h). Hence, strong and long-term UV irradiation may cause the decomposition and demonstrate the moderate photostability of this compound.

### 2.2. Photoluminescent Properties

#### 2.2.1. Solvochromic Photoluminescence

**Figure 4:**
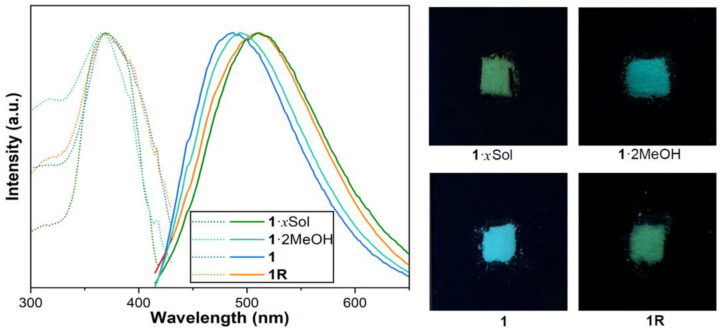
(Left) Excitation (dotted lines) and emission (solid lines) spectra of **1**·*x*Sol, **1**·2MeOH, **1**, and **1R** and (right) photos of these compounds under 365 nm excitation.

The interconversions of **1**·*x*Sol, **1**·2MeOH, **1**, and **1R** brought about visible changes in solid-state photoluminescence. As shown in Figure 4, these compounds exhibited a similar maximum excitation wavelength (*λ*_ex_) of 367 nm at room temperature (298 K), while the maximum emission wavelengths (*λ*_em_) were at 510 nm (**1**·*x*Sol and **1R**), 494 nm (**1**·2MeOH), and 486 nm (**1**). The maximum *λ*_ex_ and *λ*_em_ of **1**·*x*Sol and **1R** were similar, which supports the earlier conclusion from X-ray diffraction analysis that these compounds have the same structure. The quantum yields (QYs) and emission lifetimes (τ, Figure S6) were an unmeasured QY and 3.51 ns (**1**·*x*Sol, QY not measured because this complex loses MeOH quickly in air), 2.7% and 10.96 ns (**1**·2MeOH), and 2.3% and 10.64 ns (**1**).

**Figure 5:**
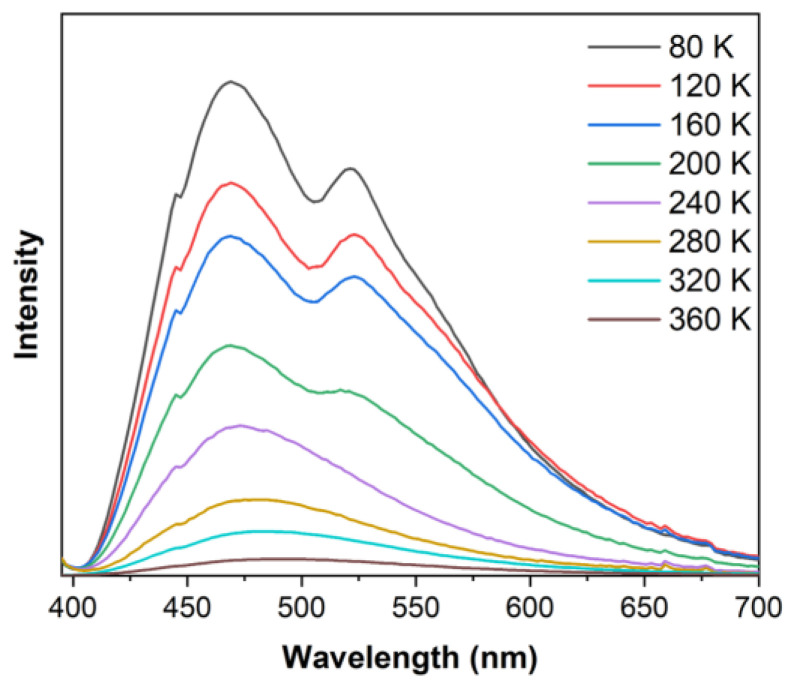
Temperature-dependent emission spectra of **1**·2MeOH from 80 K to 360 K with 40 K temperature interval (λ_ex_ = 373 nm).

The relatively short lifetimes indicated that the emissions were due to fluorescence, which is rare in Ag(I) compounds. To further investigate this photoluminescence behavior, we measured the temperature-dependent emission spectra of **1**·2MeOH. As shown in Figure 5, **1**·2MeOH exhibited typical dual emission in the lower temperature range of 80–200 K. The maximum emission wavelengths at 80 K were 465 nm (high energy band (HE), τ = 12.28 ns) and 516 nm (low energy band (LE), τ = 10.37 ms). The long emission lifetime of the LE band indicated the existence of phosphorescence at lower temperatures. This band gradually weakened and finally disappeared at higher temperatures above 240 K. The lifetime τ of the HE band at 80 K was close to that at 298 K, while the wavelength at 80 K slightly red-shifted (29 nm) when the temperature rose to 298 K. We, therefore, speculate that, because the reverse intersystem crossing (RISC) from T_1_ to S_1_ states was partly inhibited at lower temperatures [42], the phosphorescent LE band that related to the T_1_→S_0_ transition was detectable. As the temperature increased, the RISC was promoted and thus brought about the vanish of the LE band, leaving only the fluorescent HE band that related to the S_1_→S_0_ transition. Therefore, the photoluminescent behavior of **1**·2MeOH was supposed to be a TADF process [43].

**Figure 6:**
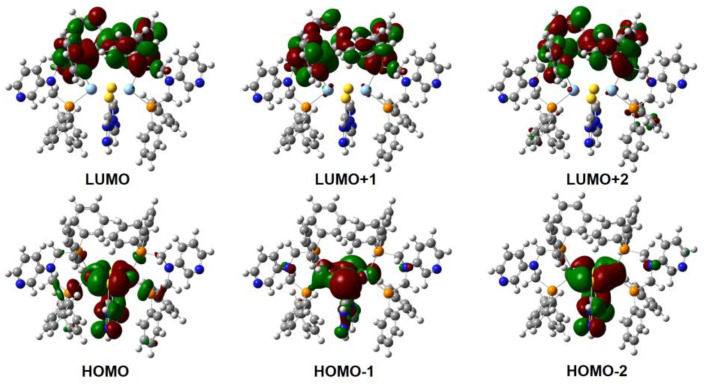
The distribution of HOMOs and LUMOs in **1**·*x*Sol.

Time-dependent density functional theory (TD-DFT) calculations based on the SCXRD data of **1**·*x*Sol were used to calculate the frontier orbital distributions. As shown in Figure 6, the HOMOs primarily consisted of the *d* orbitals of Ag(I) atoms (along the Ag-S bond direction) and the π orbital of the triazole ring, while the LUMOs distributed over the π* orbitals of the −Ph groups in 3-bdppmapy, which suggested that excitation was the mixed metal-to-ligand charge transfer (MLCT) and ligand-to-ligand charge transfer (LLCT) [44,45,46]. Calculations based on **1**·2MeOH and **1** were not performed because of a lack of SCXRD data. Attempts to change the number of MeOH molecules did not give satisfactory results. Therefore, the calculations could not give the difference in HOMO–LUMO gaps between **1**·*x*Sol, **1**·2MeOH, and **1**. 

The IR band (Figure S11) at 1679 cm^−1^ (TZ^−^) in the spectrum of **1**·*x*Sol was slightly split for **1**·2MeOH and for **1**. The bands at 1568 cm^−1^ (−Ph) and 1588 cm^−1^ (−Py) in **1**·*x*Sol slightly shifted to 1565 cm^−1^ and 1581 cm^−1^ in **1**·2MeOH and **1** and to 1564 cm^−1^ and almost absent in **1**, respectively. These changes are consistent with the elimination of solvent molecules and the cracking of voids, giving rise to additional intermolecular π···π interactions among the −Ph, −Py, and triazine rings. We propose that the blue shift of the emission from **1**·*x*Sol (510 nm) to **1**·2MeOH (494 nm) and **1** (486 nm) was caused by the variation of the energy levels of the excited and ground states that was affected by these intermolecular interactions.

#### 2.2.2. Mechanochromic Photoluminescence

**Figure 7:**
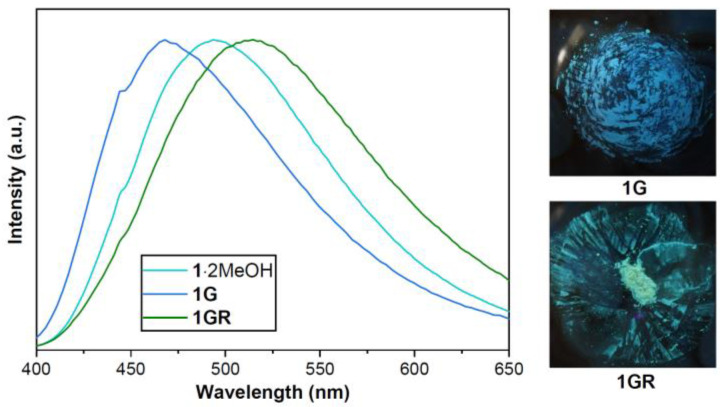
(**Left**) Emission spectra of **1**·2MeOH, **1G,** and **1GR** under 367 nm excitation. (**Right**) Photos of **1G** and **1GR** under 365 nm LED irradiation.

Grinding **1**·2MeOH with a mortar for 2 min in air resulted in white-powder **1G**, which emitted dark blue at 468 nm (Figure 7). **1G** could also be prepared with a similar treatment using **1** as the starting material. When **1G** was treated with MeOH vapor, the resulting solid **1GR** emitted green at 513 nm. Repeating this mechanical grinding and MeOH vapor treatment cycle five times led to reproducible chromic changes associated with the interconversion of **1G** and **1GR** (Figure S12).

The IR spectra of **1G** and **1GR** were similar to those of **1** and **1**·*x*Sol, respectively (Figure S11). The PXRD pattern of **1G** (Figure S13) implied it was totally amorphous, while that of **1GR** was similar to that of **1**·*x*Sol. The TGA measurement of **1G** found no weight loss below 150 °C (Figure S1), whereas that of **1GR** showed a weight loss of 3.3% at the same temperature, consistent with the loss of 1.5 MeOH (Calcd 3.3%) for each molecule of **1**. Thus, we propose that the structure of **1G** is similar to that of **1**, although there is some difference in their respective TGA curves at higher temperatures (>320 °C). We hypothesize that the mechanical forces of grinding disrupted the hydrogen bonds between the complex and MeOH solvate, leading to the elimination of MeOH molecules and the loss of crystallinity. Moreover, the unit cell of **1GR** could be restored to that of **1**·*x*Sol upon treatment with MeOH vapor, resulting in similar PXRD patterns and IR spectra. However, the binding and stoichiometry of the solvate MeOH molecules were different than those of **1**·2MeOH. Therefore, the emission wavelength of **1G** (468 nm) was different than that of **1** (486 nm), while that of **1GR** (513 nm) was quite close to that of **1**·*x*Sol (510 nm) and significantly different from that of **1**·2MeOH (494 nm).

### 2.3. Photoluminescent Sensing of Alcohols

**Figure 8:**
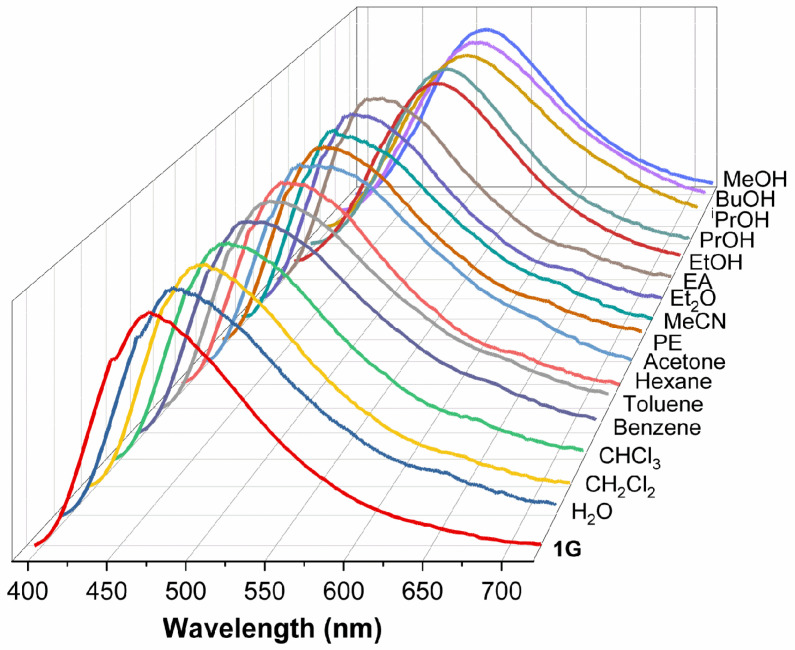
The emission spectra of **1G** upon exposure to solvent vapors (PE: petroleum ether; MeCN: acetonitrile; EA: ethyl acetate; PrOH: propanol; ^i^PrOH: iso-propanol).

The conversion of **1G** to **1GR** was accompanied by a relatively large shift in emission wavelength (45 nm) that was discernible to the naked eye, and so this interconversion was investigated for the selective sensing of alcohols. As shown in Figure 8, when **1G** was exposed to water vapor or to the vapor of common organic compounds for 5 min, its emission wavelength remained essentially the same, except when the vapor was saturated with a low–molecular weight alcohol. We suggest that only alcohols are capable of interacting with **1G** to promote the conversion to **1GR** and accompanying photochromism. Other molecules either lack the −OH group or are too small (e.g., H_2_O) to expand the distance between **1G** molecules.

**Figure 9:**
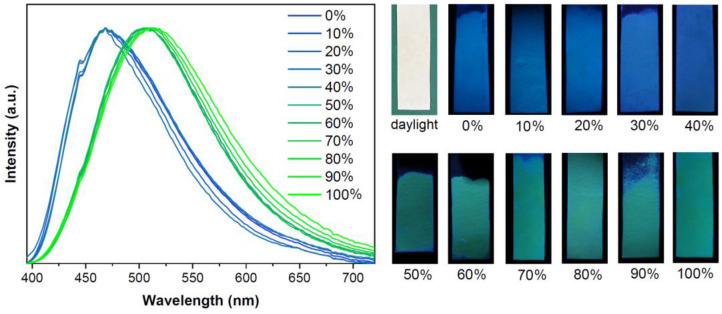
(**left**) Emission spectra of **1G** after exposure to mixed MeOH/H_2_O vapors of different MeOH content (λ_ex_ = 367 nm). (**right**) Test papers under natural daylight and under 365 nm UV light after exposure to MeOH/H_2_O vapors.

Solids of **1G** were exposed to vapors from aqueous MeOH solutions of different concentrations, and their emission spectra were recorded (Figure 9, left). The emission wavelength of **1G** after treatment remained unchanged when the aqueous mixture contained 40% (*v*/*v*) or less MeOH but shifted to 505–513 nm for concentrations above 50%. We, therefore, prepared test papers containing finely ground **1G**, which was colorless in natural daylight and emitted blue under 365 nm UV light. The emission color changed from blue to green when treated with vapors of aqueous MeOH containing more than 50% MeOH, indicating this test paper could serve as a crude sensor for the detection of MeOH in water.

## 3. Experimental Section

### 3.1. Materials, Characterization, and Measurements

3-Bdppmapy was prepared using a method from the literature [47]. All other materials were supplied from commercial sources and used as received. Elemental analyses (EAs) were performed on a Carlo-Erba CHNO-S microanalyzer. Powder X-ray diffraction (PXRD) patterns were recorded on a Bruker D2 Phaser X-ray diffractometer with a Cu *Kα* source (30 kV, 10 mA). IR spectra were acquired on a Bruker VERTEX 70+ HYPERION 2000 spectrometer. Thermogravimetric analysis (TGA) data were performed on a TA SDT2960 analyzer from room temperature to 500 °C under a nitrogen atmosphere, with a heating rate of 10 °C/min. NMR spectra were recorded on a Varian UNITY plus-400 MHz NMR instrument at ambient temperature. Photoluminescence measurements, transient photoluminescence, and quantum yield measurements were conducted using an FLS-1000 (Edinburgh Instruments, Livingston, UK) spectrometer.

### 3.2. Synthesis

#### 3.2.1. Synthesis of **1**·*x*Sol

CF_3_COOAg (26.4 mg, 0.12 mmol) and HTZ (12 mg, 0.12 mmol) were added to a mixed CH_2_Cl_2_/MeOH (*v*/*v* = 2:1, 6.0 mL) solvent, and the solution was stirred for 1 h at ambient temperature. 3-Bdppmapy (56.1 mg, 0.12 mmol) was added with further stirring over 2 h. The resulting solution was filtered and diffused with hexane. Colorless block crystals of [Ag_2_(TZ)_2_(3-bdppmapy)_2_]·*x*Sol (**1**·*x*Sol) formed in 5 days. Yield: 105 mg (95% based on Ag).

#### 3.2.2. Synthesis of **1**·2MeOH

**1**·2MeOH was prepared by evacuating **1**·*x*Sol in air for two days or under vacuum for 12 h at room temperature, while **1** was produced via vacuum treatment for 6 h at 70 °C. The yields were almost quantitative. Anal. Calcd for C_68_H_68_Ag_2_N_10_O_2_P_4_S_2_: C 55.90, H 4.69, N 9.59. Found C 55.26, H 4.10, N 9.04. IR (ATR, cm^−1^): 3355 (w), 3053 (w), 2932 (w), 2824 (w),1687 (m), 1666 (m), 1581 (m), 1565 (m), 1485 (m), 1433 (m), 1376 (m), 1275 (w), 1247 (w), 1226 (m), 1196 (m), 1129 (w), 1096 (s), 966 (w), 848 (m), 794 (m), 741 (s), 694 (s). ^1^H NMR (400 MHz, DMSO-*d_6_*, 298 K, ppm) *δ* 7.92 (s, 3H), 7.51 (s, 18H), 7.34 (t, *J* = 7.4 Hz, 11H), 7.16 (t, *J* = 7.4 Hz, 16H), 6.54 (s, 4H), 4.85 (s, 8H), 3.50 (s, 6H). ^13^C NMR (101 MHz, DMSO-*d_6_*, 298 K, ppm) *δ* 167.4, 158.5, 158.2, 144.4, 133.6, 132.1, 130.8, 129.1, 122.8, 119.3, 116.3, 55.6, 14.4. ^31^P NMR (162 MHz, DMSO-*d_6_*, 298 K, ppm) *δ* −9.19.

#### 3.2.3. Synthesis of **1**

**1**·2MeOH (20 mg) was placed in a glass tube, which was heated in a 70 °C oil bath and kept under vacuum for 3 h. The resulting solid **1** was then collected. Anal. Calcd for C_66_H_60_Ag_2_N_10_P_4_S_2_: C 56.74, H 4.33, N 10.02. Found C 56.40, H 4.32, N 9.78.

#### 3.2.4. Synthesis of **1G**

**1**·2MeOH (10 mg) was ground in a mortar in air at ambient temperature for 2 min. The resulting solid **1G** was carefully collected for further experiments. Anal. Calcd for C_66_H_60_Ag_2_N_10_P_4_S_2_: C 56.74, H 4.33, N 10.02. Found C 55.74, H 4.22, N 9.69.

#### 3.2.5. Synthesis of **1GR**

A small beaker (5 mL) containing **1G** (10 mg) was placed in a 50 mL beaker containing 10 mL MeOH, which was then sealed and left at ambient temperature for more than 0.5 h to accomplish the change. The resulting solid **1GR** was then collected directly from the beaker. Anal. Calcd for C_67.5_H_66_Ag_2_N_10_O_1.5_P_4_S_2_: C 56.10, H 4.60, N 9.69. Found C 55.95, H 4.49, N 9.85. 

### 3.3. Solvochromic Experiments of **1G** toward VOCs and H_2_O

**1G** (1 mg) was coated on a quartz slide and then sealed in a quartz cell with a small tube containing 0.5 mL solvent or water. The emission spectra were then recorded after 5 minutes of exposure.

### 3.4. Preparation of the Test Paper

**1G** (6 mg) was finely ground and then suspended in Et_2_O (3 mL) with sonication. The mixture was dropped onto filter paper strips (1 × 5 cm^2^), which were left to dry in air.

### 3.5. Single-Crystal X-ray Diffraction (SCXRD) Determination of **1**·xSol

A single crystal of **1**·*x*Sol was selected directly from the synthesis. The diffraction data were collected on a Bruker Apex-II diffractometer using graphite-monochromated Mo *Kα* (λ = 0.71073 Å) radiation at 120 K. The data were reduced by Bruker SAINT [48], while an adsorption correction (multi-scan) was applied using SADABS-2016/2 [49]. The structure was solved using direct methods and refined using full-matrix least-squares methods against *F*^2^ by SHELXL-2016/6 (Sheldrick, 2016) [50]. All non-hydrogen atoms were refined anisotropically. The hydrogen atoms of the −OH groups of MeOH were first located from a Fourier map and then refined to ride on the O atoms. All other hydrogen atoms were added in idealized positions and constrained to ride on their parent atoms. The unlocated solvent molecules were analyzed by the SQUEEZE (in PLATON v1.18) program. A summary of the key crystallographic data is given in Table 2. Selected bond lengths and angles are listed in Table S1.

### 3.6. TD-DFT Computational Details

Computational investigations were carried out using the Gaussian 16 (Rev C.01) software package using DFT and TD-DFT methods [51,52,53]. The CIF data of the crystal structure of **1**·*x*Sol was used as the initial structure. The heavy atoms were frozen, and the H atoms were optimized at the PBE0-GD3BJ/def2SVP level [54]. The above optimization results were used to calculate the excited states of S0-S50 using the TD-DFT method at the PBE0-GD3BJ/def2TZVP level [55,56].

## 4. Conclusions

We synthesized a dinuclear Ag/P/S complex [Ag_2_(TZ)_2_(3-bdppmapy)_2_]·*x*Sol (**1**·*x*Sol). The gradual loss and rebinding of the solvent molecules resulted in reversible interconversions between **1**·*x*Sol, **1**·2MeOH, and **1**, accompanied by solid-state photoluminescent emission changes. Grinding solids of **1**·2MeOH resulted in the formation of **1G**, which gave **1GR** in MeOH vapor. The interconversion of **1G** and **1GR** was reversible, as indicated by PXRD analysis and an associated emission color change between blue and green. Complex **1G** could be used as a selective sensor for the detection of alcohols. A test paper was prepared to detect MeOH vapor from aqueous mixtures. We propose that this solvochromic and mechanochromic photoluminescent behavior could be attributed to a change of intermolecular interactions that affected the energy levels of the excited and ground states during the mixed MLCT and LLCT process. This work provides an interesting example of multiple stimuli–responsive chromic photoluminescent Ag(I) complexes involving the introduction of phosphine and thiolate ligands.

## Figures and Tables

**Table 1:** Hydrogen bond lengths (Å) and angles (°) in **1**·*x*Sol.

D−H···A	D−H	H···A	D···A	D−H···A
N3−H3N···O1	0.88	1.89	2.739(6)	160.3
N8−H8N···O2	0.88	1.92	2.771(6)	161.9
O1−H1A···N1 ^i^	0.85	1.88	2.726(5)	174.7
O2−H2A···N6 ^ii^	0.85	1.86	2.711(5)	178.0

Symmetry codes for ^i^: 1-x, 1-y, 1-z. Symmetry codes for ^ii^: 1-x, -y, 2-z.

**Table 2:** Selected crystallographic data and refinement parameters for **1**·*x*Sol.

Compound	1·*x*Sol
Empirical formula	C_68_H_68_Ag_2_N_10_O_2_P_4_S_2_
Formula weight	1461.06
Crystal system	Triclinic
Space group	*P*ī
*a*/Å	16.3659(15)
*b*/Å	17.0033(15)
*c*/Å	17.3911(15)
*α*/°	107.557(2)
*β*/°	105.421(2)
*γ*/°	101.808(3)
Volume/Å^3^	4228.9(7)
Z	2
*ρ*_calc_ g/cm^3^	1.147
*μ*/mm^−1^	0.629
*F*(000)	1496
*R*_1_ ^a^	0.0621
*wR*_2_ ^b^	0.1846
GOF ^c^	1.052

^a^ *R*_1_ = Σ||*F*_o_|−|*F*_c_||/Σ|*F*_o_|. ^b^ *wR*_2_ = {Σ*w*(*F*_o_^2^ − *F*_c_^2^)^2^/Σ*w*(*F*_o_^2^)^2^}^1/2^. ^c^ GOF = {Σ*w*((*F*_o_^2^ − *F*_c_^2^)^2^)/(*n* − *p*)}^1/2^, where *n* is the number of reflections and *p* is the total number of parameters refined.

## Data Availability

The crystallographic data are available from the Cambridge Crystallographic Data Centre (CCDC number 2266377). Other data not presented in the Supplementary Materials are available on request from the corresponding author.

## Supplementary Materials

The following supporting information can be downloaded at https://www.mdpi.com/article/10.3390/molecules28145513/s1. Table S1: Selected bond lengths (Å) and angles (°) for **1**·*x*Sol; Figure S1: TGA curves of **1**·*x*Sol, **1**·2MeOH, **1**, **1R**, **1G,** and **1GR** under N_2_ atmosphere; Figure S2: IR spectra of **1**·2MeOH and HTZ; Figures S3–S5: ^1^H, ^13^C, and ^31^P NMR spectra of **1**·2MeOH; Figures S6–S8: Transient photoluminescent data for **1**·*x*Sol, **1**·2MeOH, and **1** at ambient temperature; Figures S9 and S10: Transient photoluminescent data for **1**·2MeOH at 80 K; Figure S11: IR spectra of **1**·*x*Sol, **1**·2MeOH, **1**, **1G**, **1GR,** and 3-bdppmapy in the region of 1800–1300 cm^−1^; Figure S12: Emission wavelength of **1G**/**1GR** upon exposure to MeOH vapor and grinding over five cycles; Figure S13: PXRD patterns of **1**·*x*Sol, **1**·2MeOH, **1G,** and **1GR**.
