# Supplementary material for: A Multiple Stimuli–Responsive Ag/P/S Complex Showing Solvochromic and Mechanochromic Photoluminescence
Source: Molecules. 2023 Jul 19;28(14):5513. doi: 10.3390/molecules28145513 (PMC10384712; doi:10.3390/molecules28145513)
Supplement: Supplementary file 1 [file molecules-28-05513-s001.zip › molecules-2461757-supplementary.pdf]

Contents

Table S1. Selected bond lengths (Å) and angles (°) for **1**·*x*Sol.

Figure S1. TGA curves of **1**·*x*Sol, **1**·2MeOH, **1**, **1R**, **1G** and **1GR** under N_2 atmosphere.

Figure S2: IR spectra of **1**·2MeOH and HTZ.

Figure S3. ^{1}H NMR spectrum of **1**·2MeOH.

Figure S4. ^{13}C NMR spectrum of **1**·2MeOH.

Figure S5. ^{31}P NMR spectrum of **1**·2MeOH.

Figures S6. Transient photoluminescent data for **1**·*x*Sol at ambient temperature.

Figures S7. Transient photoluminescent data for **1**·2MeOH at ambient temperature.

Figures S8. Transient photoluminescent data for **1** at ambient temperature.

Figure S9. Transient photoluminescent data for **1**·2MeOH (λ_{ex} = 373 nm, λ_{em} = 465 nm) at 80 K.

Figure S10. Transient photoluminescent data for **1**·2MeOH (λ_{ex} = 373 nm, λ_{em} = 516 nm) at 80 K.

Figure S11. IR spectra of **1**·*x*Sol, **1**·2MeOH, **1**, **1G**, **1GR** and 3-bdppmapy in the region of 1800-1300 cm^{-1}.

Figure S12. Emission wavelength of **1G/1GR** upon exposure to MeOH vapor and grinding over five cycles.

Figure S13. PXRD patterns for **1**·*x*Sol, **1**·2MeOH, **1G** and **1GR**.

Table S1. Selected bond lengths (Å) and angles (°) for **1**·*x*Sol.

Ag**1**–Ag2	3.3076(5)	S1–Ag1–S2	99.57(3)
Ag1–S1	2.6388(10)	S1–Ag1–Ag2	50.71(2)
Ag1–S2	2.5856(10)	S1–Ag2–S2	99.93(3)
Ag1–P1	2.4667(10)	S1–Ag2–Ag1	51.29(2)
Ag1–P2	2.4723(10)	S2–Ag1–Ag2	50.41(2)
Ag2–S1	2.6173(10)	S2–Ag2–Ag1	50.21(2)
Ag2–S2	2.5932(10)	P1–Ag1–Ag2	117.15(3)
Ag2–P3	2.4552(11)	P2–Ag1–Ag2	140.88(3)
Ag2–P4	2.4716(10)	P3–Ag2–Ag1	116.76(3)
		P4–Ag2–Ag1	141.04(3)

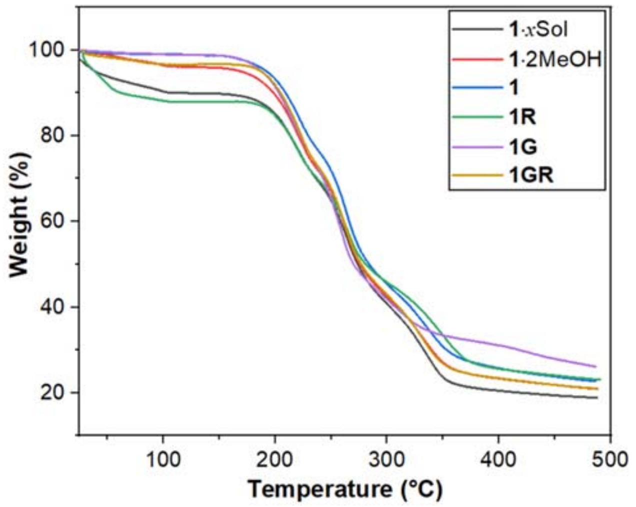

Figure S1. TGA curves of **1**·*x*Sol, **1**·2MeOH, **1**, **1R**, **1G** and **1GR** under a N_2 atmosphere.

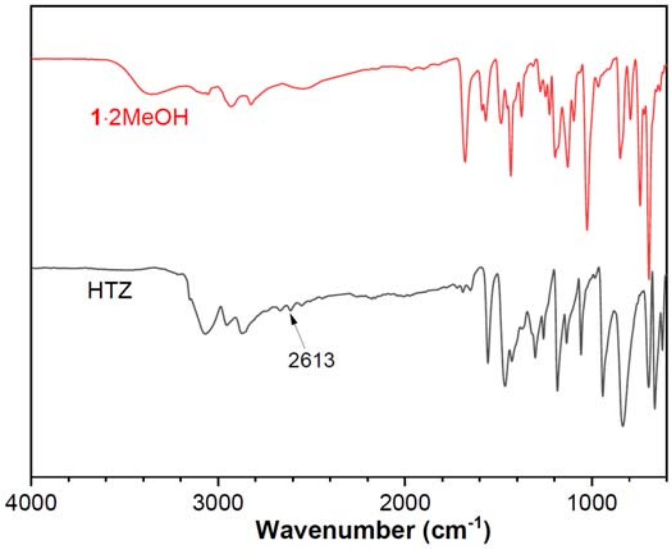

Figure S2. IR spectra of **1**·2MeOH (red) and HTZ (black).

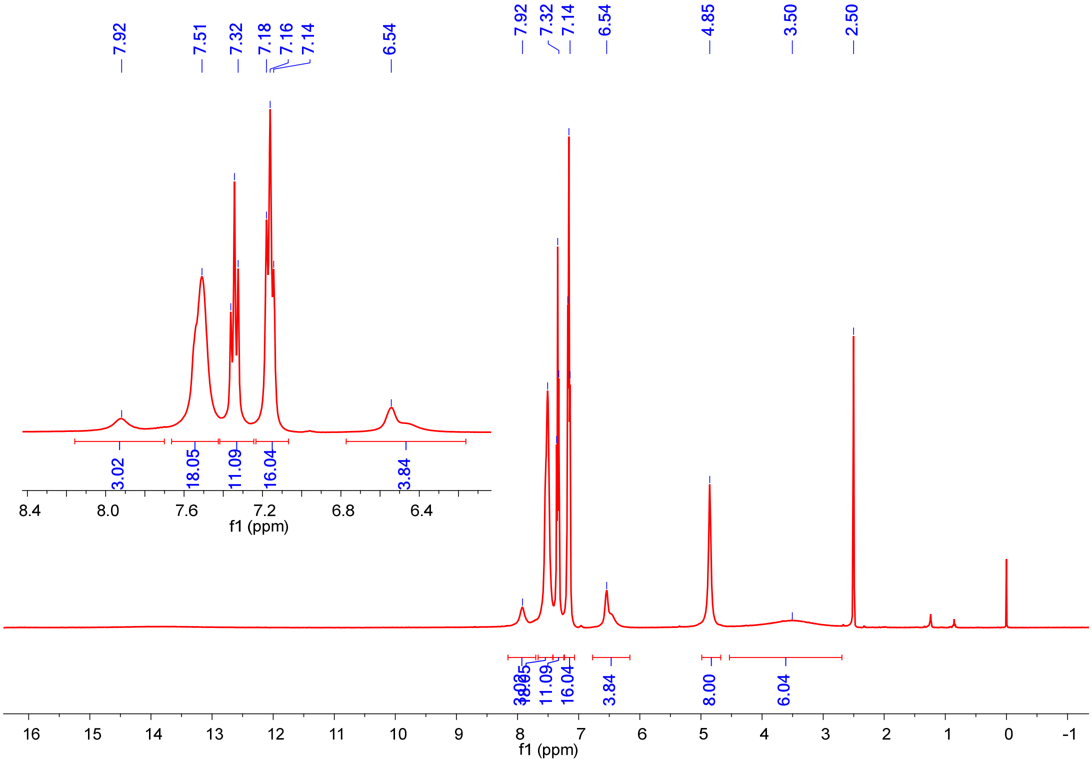

Figure S3. ^{1}H NMR spectrum of **1**·2MeOH.

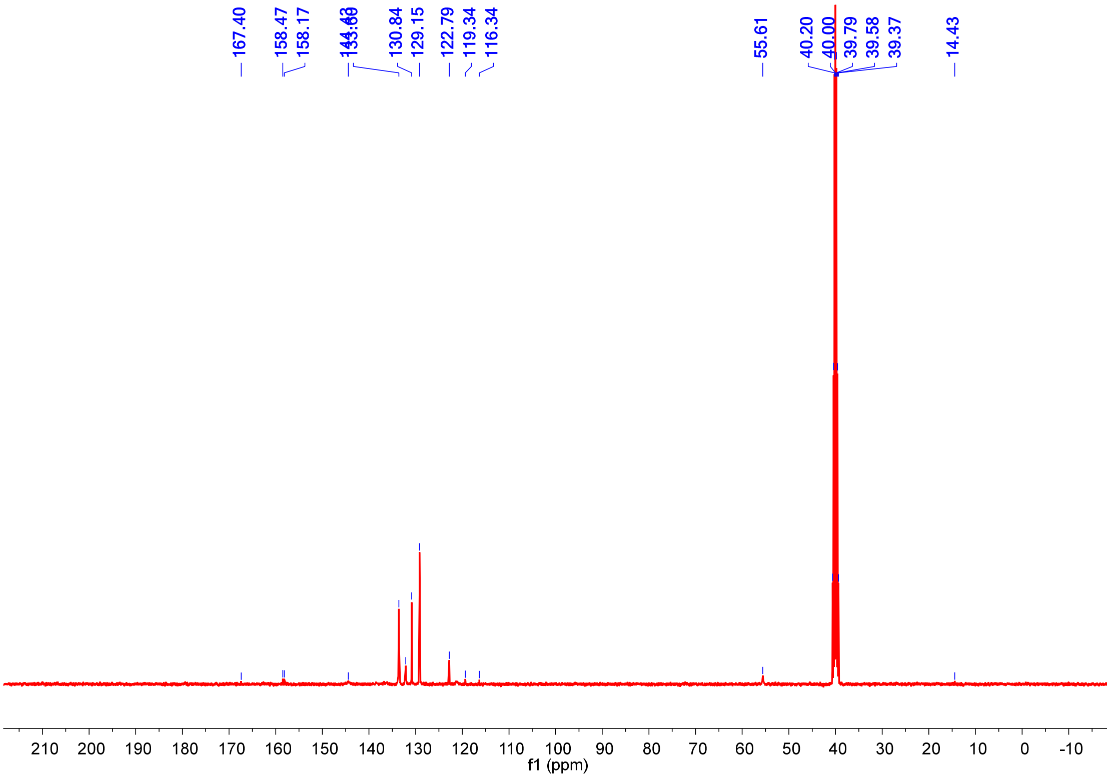

Figure S4. ^{13}C NMR spectrum of **1**·2MeOH

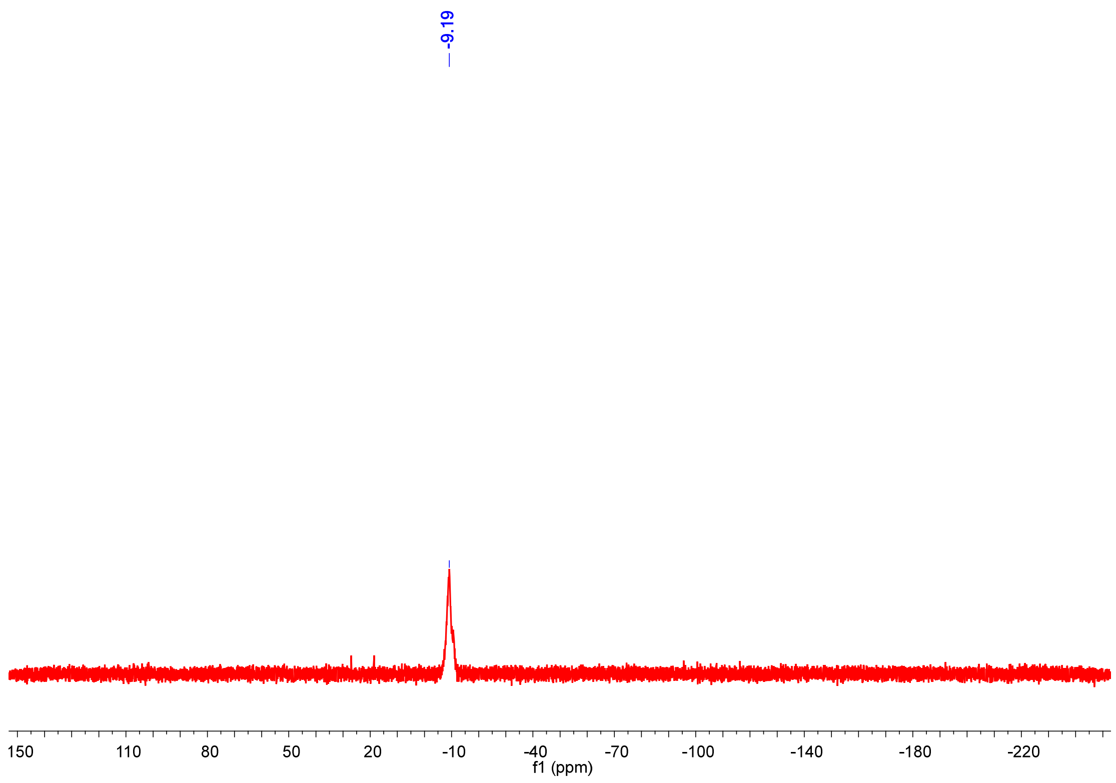

Figure S5. ^{31}P NMR spectrum of **1**·2MeOH

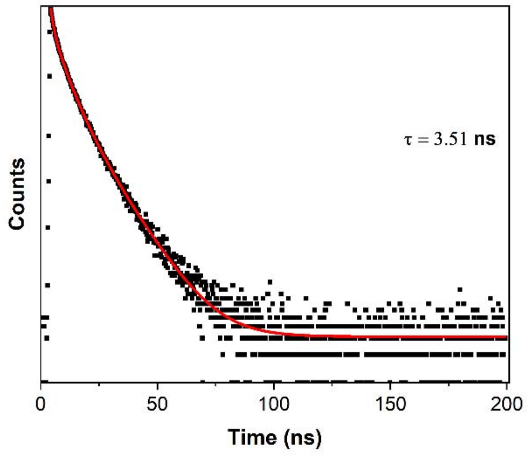

Figure S6. Transient photoluminescent data for **1**·xSol (λ_{ex} = 373 nm) at ambient temperature.

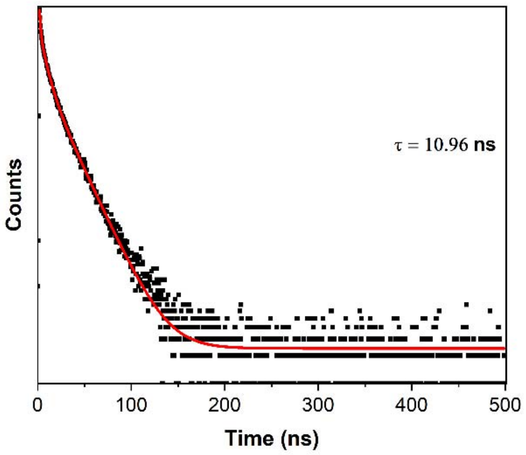

Figure S7. Transient photoluminescent data for **1**·2MeOH (λ_{ex} = 373 nm) at ambient temperature.

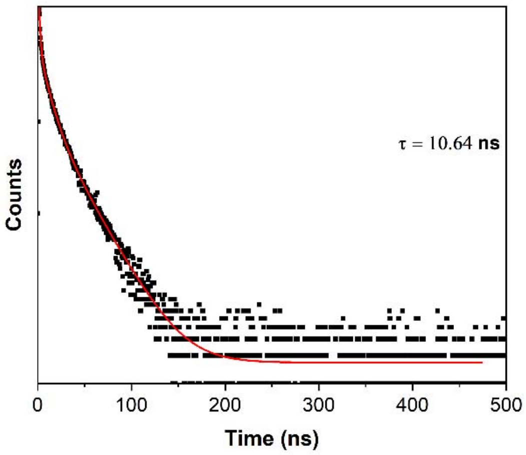

Figure S8. Transient photoluminescent data for **1** (λ_{ex} = 373 nm) at ambient temperature.

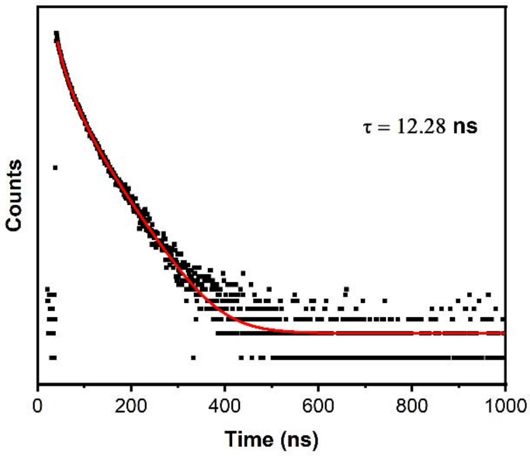

Figure S9. Transient photoluminescent data for **1**·2MeOH (λ_{ex} = 373 nm, λ_{em} = 465 nm) at 80 K.

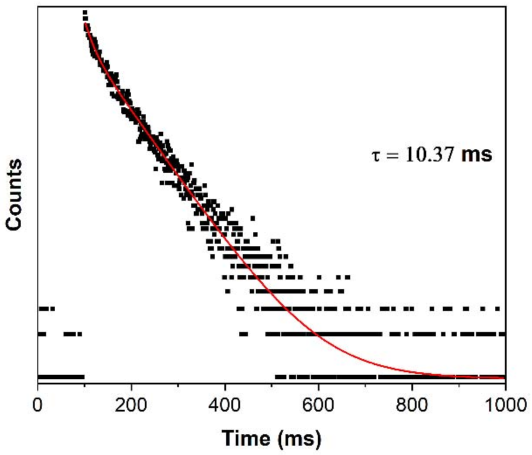

Figure S10. Transient photoluminescent data for **1**·2MeOH (λ_{ex} = 373 nm, λ_{em} = 516 nm) at 80 K.

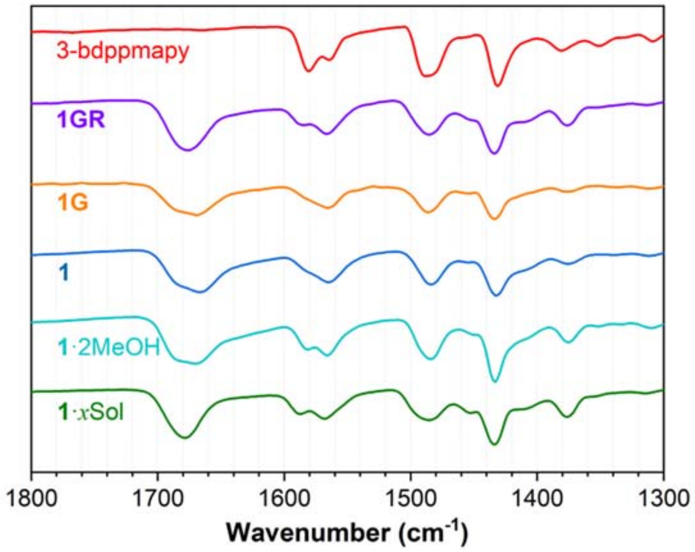

Figure S11. IR spectra of **1**·*x*Sol, **1**·2MeOH, **1**, **1G**, **1GR** and 3-bdppmapy in the region of 1800-1300 cm^{-1}.

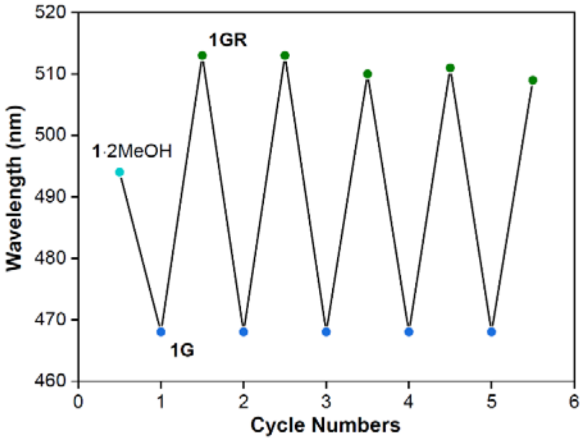

Figure S12. Emission wavelength of **1G**/**1GR** upon exposure to MeOH vapor and grinding over five cycles.

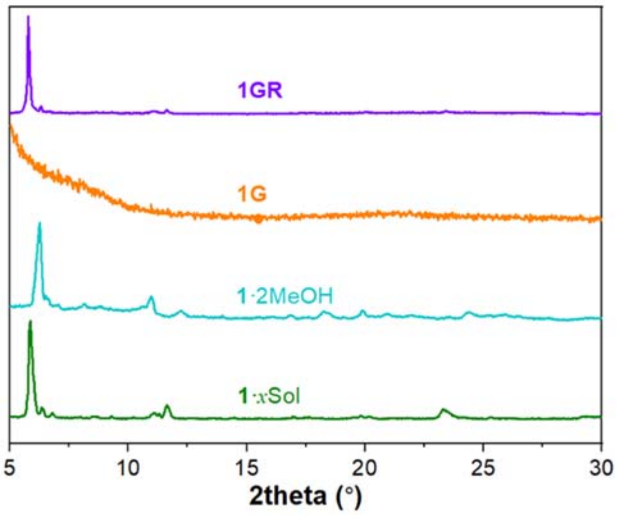

Figure S13. PXRD patterns for **1**·*x*Sol, **1**·2MeOH, **1G** and **1GR**.
